# High levels of anxiety and depression in diabetic patients with Charcot foot

**DOI:** 10.1186/1757-1146-7-22

**Published:** 2014-03-21

**Authors:** Zahra Chapman, Charles Matthew James Shuttleworth, Jörg Wolfgang Huber

**Affiliations:** 1 Department of Foot Health, Mile End Hospital, Barts and London Trust, Bancroft Road, London E1 4DG, UK; 2 Community Mental Health Team, Surrey and Borders Partnership Foundation NHS Trust, Randalls Park Drive, Leatherhead KT22 7AD, UK; 3 Centre for Health & Wellbeing Research, University of Northampton, Northampton, UK, now Centre for Health Research, University of Brighton, Falmer, Brighton BN1 9PH, UK

**Keywords:** Charcot foot, Diabetic foot, Diabetes, Depression, Anxiety

## Abstract

**Background/aims:**

Charcot foot is a rare but devastating complication of diabetes. Little research is available on the mental health impact of Charcot foot. Aim of the study is to assess mental health in diabetes patients with Charcot foot and to investigate the moderating effects of socio-demographic factors. The severity of the problem will be statistically evaluated with the help of a reference data set.

**Methods:**

Cross-sectional questionnaire data using the Hospital Anxiety and Depression Scale (HADS) and demographic background were collected from 50 patients with diabetes and Charcot complications (males 62%; mean age 62.2 ± 8.5 years). Statistical comparisons with a large data set of general diabetes patients acting as a point of reference were carried out.

**Results:**

Anxiety and depression levels were high, (anxiety and depression scores 6.4 ± 4 and 6.3 ± 3.6 respectively). Females reported more severe anxiety and depression. Ethnic minorities and patients out of work reported more severe anxiety. Comparisons with published HADS data indicate that diabetes patients with Charcot foot experience more serious levels of anxiety and depression.

**Conclusions:**

The high levels of mental health problems which were found in this study in diabetes patients with Charcot foot require recognition by researchers and clinicians. The findings imply the need to screen for mental health problems in diabetes patients with Charcot foot.

## Introduction

Charcot foot is a rare but potentially devastating complication of diabetes mellitus [1]. Despite an extensive literature linking diabetes and psychological morbidity, the clinical management of diabetic foot problems does not focus on psychological issues.

Charcot foot, also known as ‘neuroarthropathy/neuropathic osteoarthropathy’, ‘Charcot joint’ [2] or ‘neurogenic osteoarthropathy’ [3], is a disease of bone that is chronic and progressive, and results in destruction of joints or bones which have lost sensation [2]. Loss of protective sensory innervation causes neuropathic changes which can render the condition either painful or painless [4]. No universally accepted criteria for the diagnosis of Charcot foot are available [5]. Patients may present with inflammation of the foot or ankle [6] which may have been triggered by traumatic injury despite no obvious structural damage. The joint area is usually warm and the temperature elevated by between 3 to 6° Celsius [7]. In the first three weeks of acute Charcot foot there are normally no changes seen on X-ray [7]. During later stages of Charcot foot radiographic investigation may reveal some evidence of dislocation or fracture of the foot [8].

Diabetes mellitus and depression are recognised as two of the most important public health issues in the UK and elsewhere. Major reviews of cross-sectional data argue that as a rule of thumb diabetes increases the risk of depression 2-fold compared to those without diabetes [9,10]. However, absolute figures vary as function of type of assessment with questionnaire studies reporting twice the prevalence compared to studies based on psychiatric interviews. This difference has also been observed in non-diabetic populations; the prevalence of depressive disorders in non-diabetic comparison groups, as assessed by full diagnostic interview, has been reported as 5% in a meta-analytic review (9). Both diabetes and depression have profound consequences for individuals and society [11]. Anxiety can manifest itself in a variety of overlapping neurotic disorders including generalised anxiety disorder, phobias, obsessive-compulsive disorder, post-traumatic stress disorder and panic disorder [12]. Anxiety symptoms are more common in patients with diabetes compared to those without [13].

Psychological comorbidity confers additional risks on patients with diabetes resulting in poorer self-care and poorer outcomes [14]. Depression in type 2 diabetes has been shown to be associated with twice the rate of a first diabetic foot ulcer over a 4 year follow-up period [15] and higher rates of amputation [16]. In addition depression in first diabetic foot ulcer is associated with a two-fold increase of mortality over 5 years [17].

In patients with diabetes, both depression and anxiety levels have been demonstrated to be higher in those with chronic foot ulceration than in controls [18]. For anxiety, it has been argued that its links with self-care and clinical diabetes outcomes are less clear compared to those of depression; dependent on the nature of the anxiety problem, poorer or even enhanced self-care may be envisaged [19].

Existing research has not focused on psychological morbidity in patients specifically with diabetic Charcot foot. Ulcerations and Charcot foot have either been grouped together [20] or Charcot foot is not mentioned as a distinct diagnosis [21]. Existing studies are based on small groups of Charcot patients, but findings specific to this group are not presented in publications [22]. Foot ulceration is much more common than Charcot foot in diabetes; thus it is not unusual to recruit samples which exclude patients with Charcot foot [15,23]. The severity of foot problems in diabetes has been addressed in qualitative research, but only in persons with a diabetic foot ulcer rather than with Charcot foot again suggesting that the latter condition has not been studied specifically in qualitative research either: *‘When you stop to think about it [the ulcer] you could just get up and go and now you can’t; you’re restricted; you have to take care not to knock your leg; you have to be careful that you don’t tread on anything that you know is on the ground.‘* In the same paper a male having lost his job consequent to ulceration is quoted: *‘I would lose my life as well. In fact I think I did.’*[24]. Whether ulceration or Charcot foot is worse, is an open question. However, it is clear that very limited data is available on mental health in patients with diabetes and Charcot foot.

Thus, the aim of the present paper is twofold: (i) to report on mental health on the basis of HADS scores (anxiety and depression symptoms) in patients with diabetes and Charcot foot and (ii) to compare our findings with HADS comparison data in patients with diabetes with and without complications. We shall report full details on our patient group with diabetes and Charcot foot, as the authors are not aware of any recent study specifically recruiting patients with diabetes and Charcot foot.

## Methods and patients

A cross-sectional questionnaire survey was carried out. A group of patients with a confirmed diagnosis of diabetes with Charcot foot were recruited from a tertiary care facility in South London (King’s College Hospital) between March 2010 and July 2010. King’s College Hospital diabetic foot clinic is a referral centre for diagnosis and treatment of Charcot foot as well as a variety of other serious foot complications. The main treatment offered for Charcot foot is a total contact cast to stabilise and off load pressure from joints and foot. All patients were recruited from the same clinic waiting area; assistance was provided as and when necessary (e.g. questions were read out to individuals with impaired vision).

Data on anxiety and depression were collected by means of the validated and widely used Hospital Anxiety and Depression Scale (HADS) [25]. A major advantage of this scale is that it carries a lower risk of confounding diabetes-related and psychological morbidity symptoms compared to some other widely used scales. The internal consistency reliabilities of the HAD sub-scales were calculated using Cronbach’s alpha coefficient. Alpha coefficients for anxiety and depression were 0.86 and 0.78 respectively, indicating good reliability. HADS data were initially analysed using standard classifications: the HADS scores for anxiety and depression were categorised into three groups: scores 0-7 “no risk”, 8-10 “borderline risk of anxiety or depression” and scores of ≥11 as intermediate or severe risk of anxiety or depressive (“intermediate risk” for short). In addition, anxiety and depression scores were dichotomised using the following cut points: a score between 0 and 7 was classed as normal and a score between 8 – 21 points as increased risk. Diabetes related information and demographics were provided by patients on a self-designed recording sheet: patient age at time of recruitment, gender, self-reported ethnicity, marital status (married/cohabiting, separated/divorced, single, other), occupational status and the foot site affected by Charcot.

The case definition for Charcot foot was: (1) diagnosis by an expert clinician with a confirmed date of diagnosis; (2) Charcot was located in the anatomical foot. Exclusion criteria were any active foot ulceration or amputation, learning disability, inability to read and write in English, pregnancy or lactation, and other significant acute or chronic illnesses. Patients currently receiving psychoactive drugs or psychiatric treatment were excluded because these treatments would be expected to considerable improve anxiety and depression levels and therefore lead to an underestimation of mental health problems in patients with Charcot foot.

Ethical approval was obtained from University of Roehampton, King’s College Hospital NHS Foundation Trust and Outer North London Research Ethics Committee (REC Ref No 10/HO724/8). Participants provided informed consent.

### Statistical analysis

Data were checked and analysed using Microsoft Excel and SPSS V20. Frequencies and percentages were calculated. Normality assumptions for the HADS scales were not violated (Kolmogorov-Smirnov test p-values >0.10). Means and standard deviations (mean ± SD) are reported for scaled data, but where the focus is on differences or the comparison with reference data (see below) we used 95% confidence intervals. We chose this form of presentation to provide the most relevant information to the reader (standard deviations and confidence intervals can be mutually transformed if relevant sample sizes are provided as we did). Anxiety and depression scores were analysed using dimensional scale scores and also as grouped data, after converting scale scores into 2 or 3 risk categories (full details in results section). Bi-variate comparisons used t-tests. Comparisons with published data from Collins et al. [26] will be used as a key point of reference as far as possible; we refer to these data as the *reference data*. Their relatively large population sample of 1456 patients (general practice and out-patients from the Republic of Ireland) includes 296 patients with type 1 diabetes; both bi- and multi-variate results do not suggest an effect of type of diabetes on anxiety or depression scores. It is therefore justified to carry out the comparisons with these reference data (this approach is supported by Perry as the senior author of the paper, personal communication, 2013) [26]. All analyses using SPSS applied bootstrap methods to minimise potential problems associated with non-normality and the relatively small Charcot patient group of n = 50. Significance was set at p ≤ 0.05; whenever statistical associations or differences are discussed, they are significant (unless stated otherwise). Full details on statistical findings are provided in tables and figures.

## Results

### Patient characteristics

The participants were predominantly male (31 males and 19 females) with a mean age of 62 years. Data on marital status, work status and ethnicity and other sample characteristics are provided in Table 1. The most common site of Charcot foot problems was the mid-foot (54%).

**Table 1:** Descriptive information for Charcot sample (n = 50) (mean ± SD; n and percentages)

**Variable**	**Mean ± SD or n (%)**	**Mean Anxiety**	**Mean Depression**
		**Score ± SD**	**Score ± SD**
Female	19 (38%)	8.4 ± 4.0**	7.6 ± 3.4^♯^
Male	31 (62%)	5.1 ± 4.2	5.6 ± 3.5
Age (years)	62.2 ± 8.5		
≤ 60 years	24 (48%)	6.8 ± 4.3	6.8 ± 3.7
61 + years	26 (52%)	6.0 ± 4.5	5.9 ± 3.5
White	38 (76%)	5.8 ± 4.3^♯^	5.7 ± 3.4*
Non—white (BME)	12 (24%)	8.3 ± 4.1	8.4 ± 3.4
Working	11 (22%)	5.8 ± 4.2	4.4 ± 4.1*
Not working	39 (78%)	6.5 ± 4.5	6.9 ± 3.3
Married	27 (54%)	6.8 ± 4.3	7.0 ± 3.5
Single or divorced	23 (46%)	5.9 ± 4.5	5.5 ± 3.5
Duration of diabetes (years)	20.6 ± 10.3		
≤ 17 years	25 (50%)	6.4 ± 4.4	6.4 ± 4.5
18 + years	25 (50%)	6.1 ± 3.7	6.6 ± 3.5
Duration of Charcot foot (years)	5.0 ± 3.4		
≤ 4 years	24 (48%)	6.7 ± 4.2	6.2 ± 3.7
5 + years	26 (52%)	6.1 ± 4.6	6.5 ± 3.5
Charcot site:			
Ankle joint only	9 (18%)	6.4 ± 3.7	6.4 ± 2.7
Mid-foot only	28 (56%)	6.0 ± 4.3	6.6 ± 3.8
Fore-foot only	8 (16%)	6.0 ± 5.5	5.6 ± 4.8
Ankle and Mid-foot (n = 3)/Mid- and fore-foot (n = 2)	5 (10%)	8.8 ± 4.8	5.8 ± 1.5

Mean ± SD for anxiety and depression scores.

^♯^p < 0.10, *p ≤ 0.05 **p ≤ 0.01.

### Mental health: anxiety and depression

Anxiety and depression scores for the total sample are presented in Table 2. Results are also presented in terms of risk categories: normal risk, borderline risk and intermediate risk which includes a small number of patients who are severe risk. In total, increased risk levels have been found for 42% of patients, both for anxiety and depression.

**Table 2:** Mental health (HADS) scores – means and risk levels

**HAD Scale**	**Mean ± SD**	**Normal score ≤7 n (%)**	**Borderline risk 8–10 n (%)**	**Intermediate risk ≥11 n (%)**
Anxiety	6.4 ± 4.4	29 (58%)	12 (24%)	9 (18%)
Depression	6.3 ± 3.6	29 (58%)	14 (28%)	7 (14%)

### Mental health and demographics

Female patients experience significantly higher levels of anxiety and depression. Full details can be found in Table 1. Age is not correlated with anxiety or depression scores (p-values >0.50). Following dichotomisation of age into those up to 60 years old and those older than 60, both anxiety and depression scores are lower for the older group, but the differences did not reach significance.

**Figure 1:**
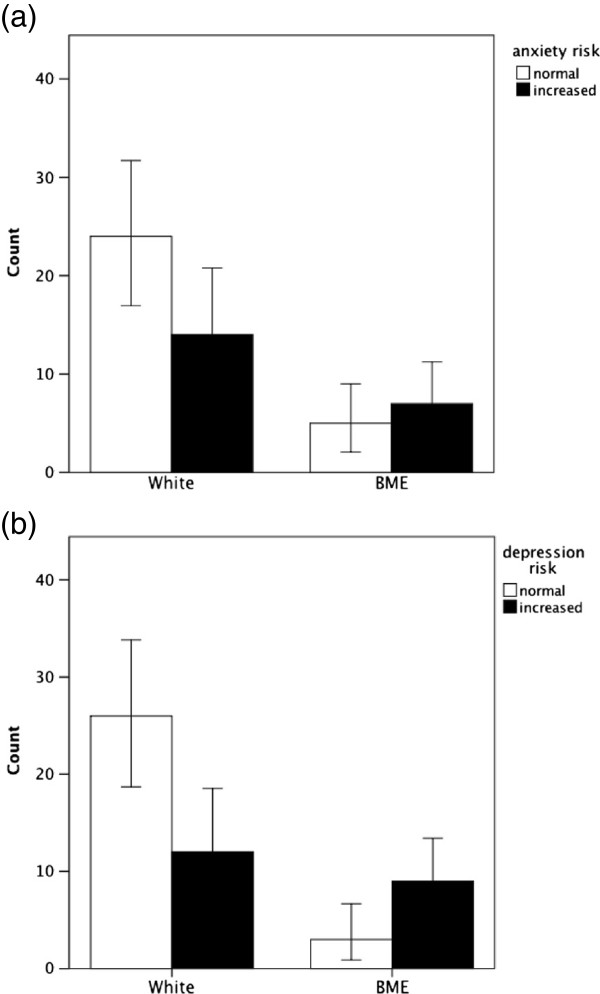
**Risks of anxiety and depression in White people with diabetes and Charcot foot compared to those from Black Minority Ethnic groups (frequency counts ± 95% CI).** The difference for anxiety risk is not significant (p ≤ 0.10), but the difference for depression is significant (p ≤ 0.05).

Due to the small numbers of black and other non-white patients, ethnicity was grouped in terms of white versus Black Minority Ethnicity (BME) patients. The number of BME patients with Charcot foot was 12 out of 50 (24%). Patients with a BME background had significantly higher depression scores, but differences for anxiety only reached borderline significance (p < 0.10) (see Figure 1). Patients in work reported lower levels of depression scores compared to those not working, but no difference was observed for anxiety scores. Marital status (married vs single or divorced) was not associated with levels of anxiety or depression scores.

### Duration of diabetes and Charcot foot

The mean duration of diabetes was 20.6 (SD = 10.3 years). The mean duration of Charcot foot was 5.0 years (SD = 3.4). Correlations between duration of Charcot foot and anxiety (r50 = 0.23, p = 0.11) and depression scores (r50 = 0.08, p = 0.59) were weak and did not reach significance. We also dichotomised these time-defined variables using the median split. Differences in anxiety and depression scores in relation to diabetes duration (up to 17 years vs longer) and Charcot foot duration (up to 4 years vs longer) were very small and did not reach significance (see Table 1).

### Comparisons with published HADS data

Results from a major study on patients with diabetes [26] using the HADS and carried out in Ireland included a sample of n = 1,456 patients with diabetes, of which 1,291 and 1,308 individuals provided anxiety and depression data respectively. Re-‒analysis of the published data produced a prevalence of 29% for increased anxiety risk and 22% for increased depression risk for type 2 diabetes patients, after excluding those with type 1. Table 3 provides estimated odds ratios and 95% confidence intervals. The odds are 1.8 for anxiety and 2.5 for depression, indicating that our sample of patients with diabetes and Charcot foot is exposed to a significantly increased risk of mental health problems compared to a large diabetes reference sample (p < 0.05).

**Table 3:** Anxiety and depression risk in the diabetes reference sample and the Charcot foot sample

		**Diabetes sample n**	**Charcot sample n**	**Odds ratio**	**CI**	**p-value**
Anxiety	Normal	779 (71%)	29 (58%)			
	Increased Risk	315 (29%)	21 (42%)	1.79	1.006 - 3.188	0.048*
De-pression	Normal	805 (78%)	29 (58%)			
	Increased Risk	233 (22%)	21 (42%)	2.50	1.401 - 4.469	0.002**

*p ≤ 0.05; **p ≤ 0.01.

**Figure 2:**
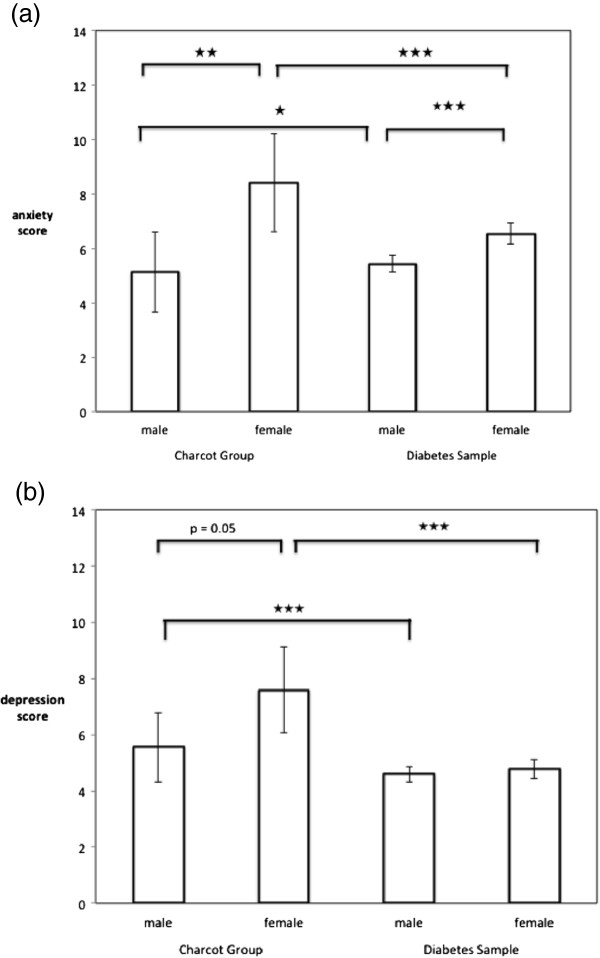
**Mean scores ± 95% confidence intervals for (a) anxiety scores and (b) depression scores.** Bars present findings for males and females for Charcot sample and diabetes reference sample. Stars indicate significance level of p based on bi-variate tests (★ p ≤ 0.05, ★★ p ≤ 0.01, ★★★ p ≤ 0.001). For full details of tests see text.

**Figure 3:**
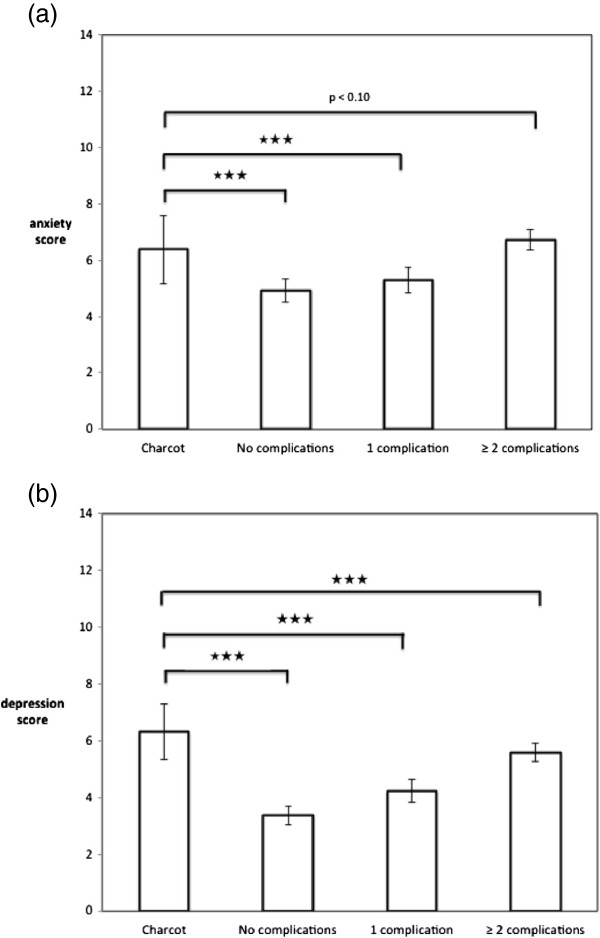
**Comparison between Charcot sample and reference data of diabetes patients without, with 1 and with 2 or more complications (diabetes sample) for (a) anxiety and (b) depression scores.** Means ± 95% CI. Stars indicate significance level of p based on bi-variate tests (★★★ p ≤ 0.001). For full details of tests see text.

Collins et al. [26] reported considerable detail regarding demographics and diabetes related characteristics. Comparisons by gender and diabetes complications were carried out. However, for this analysis the published data do not allow for the separation of type 1 and type 2 patients which is not a serious problem given that anxiety and depression levels were comparable for both groups (see also data analysis section). Comparisons of Collins et al.’s data with our Charcot sample can be found in Figure 2. Statistical results are based on one sample t-tests with Collin’s paper providing reference values. HADS scores are higher for both males and females in the Charcot foot sample compared to the reference sample data (p < 0.05). Also we compared their means and SDs for people with diabetes with no complication, one complication or 2 and more complications with our sample with Charcot foot. Figure 3 shows the results. The HADS scores for the Charcot sample are higher than for all groups in the reference sample, including those with 2 or more complications.

## Discussion

High levels of anxiety and depression scores and a high prevalence of being at risk of mental health problems were observed in our sample of 50 diabetes patients with Charcot foot. Incidentally the figures are exactly the same for anxiety and depression risks. The literature on mental health in diabetes focuses on depression and only limited data is available on anxiety levels and risks. Comparisons with findings on depression from meta--analyses of general diabetes samples [9,10] suggest that in our sample the prevalence for depression risk is double that of around 20% which is typically found for general diabetes samples.

Statistical comparisons with findings from a study which used the HADS in a large sample of diabetes patients (with and without complications) again support the view that diabetes patients with Charcot foot are at a considerably increased risk of anxiety and depression. Further comparisons with this data set on the basis of the number of complications shows that diabetes patients with Charcot foot have significantly higher depression scores than diabetes patients which have 2 or more complications. The figures for anxiety show similar patterns (P <0.10). While we did not collect data on the number of complications in our sample, the trends clearly show that patients with Charcot foot experience high levels of anxiety and depression symptoms**.** We accept that it is possible or likely that patients in this study are likely to suffer from other complications; this is typical for most studies focusing on advanced complications of diabetes. These additional complications may affect mental health and wellbeing and are of conceptual interest, but for the practitioner it is important to note that patients with Charcot foot, irrespective of the presence or absence of other complications, do suffer from very high levels of depression and to a lesser extent from increased anxiety compared to those patients from our reference sample who have 2 or more complications (26).

Demographic characteristics are linked to mental health in this sample of Charcot patients. Women suffer higher anxiety and depression levels, and members of BME groups and people out of work suffer higher depression levels, but differences for anxiety do not reach significance. Higher levels of anxiety and depression in females are in line with general trends in population samples [27]. Interestingly differences between the genders are more pronounced in the Charcot sample compared to the general diabetes sample.

Higher depression levels in BME patients and patients out of work are not unexpected and are likely to reflect greater vulnerability to mental health issues [28]. Marital status is unrelated to mental health, but it is perhaps surprising that single or divorced patients report lower, but statistically non-significant, scores. No associations were found between mental health and disease duration.

HADS data were also reported in a study by Boulton and his colleagues in Manchester (UK) [18] on patients with chronic foot ulceration and matched diabetes controls (n =13 and 26 respectively). These authors reported median scores and ranges only for the combined HAD scale (sum of anxiety and depression score): for ulceration the median = 9 (range O-16) and controls the median = 4 (O-26). We calculated the same statistic for the two groups in our study: medians for the entire HAD scale were 14 (0 – 28) for the patients with Charcot foot and 14 (0 – 34) in the comparison group. Our values are considerably higher than those observed by Boulton and colleagues (statistical significance could not be determined for this comparison, due to insufficient data provided in the paper).

This paper presents possibly the first study addressing mental health problems specifically in a sample of diabetes patients with Charcot foot. Another strong point is the use of the HADS scale which carries a lower risk of confounding symptoms of anxiety and depression with symptoms due to diabetes. The PHQ-9 questionnaire which is widely used for the assessment of depression risk, in contrast, contains items which assess symptoms common to depression and diabetes [29].

The Charcot patients were carefully identified and confounding with other acute foot problems was kept to a minimum. It needs to be remembered that Charcot foot is a rare condition with an incidence rate of 0.3% per year for a large diabetes clinic in Denmark [30]. Also all patients were recruited from the same specialist tertiary care centre, facilitating the recruitment of a sizable sample. The instrument for assessing psychological morbidity, i.e. the HADS, is one of the most widely used questionnaires for screening and risk assessment for anxiety and depression. It is accepted that a psychiatric diagnostic interview [31] would have been preferable but was not practicable for this study. Future research may also want to consider collecting more details on the history of foot problems and a detailed assessment of the severity of the current state of the Charcot foot, either by clinician judgement or using a system comparable to the Wagner or Texas classification systems [32].

There are currently no other studies available examining the mental health status in patients with Charcot foot which would allow direct comparisons with our findings. However there are two studies which have looked at health status using the SF-36 questionnaire in patients with Charcot foot [33,34]. They both found that the so-called ‘physical health component summary scores’ (PCS), which is a measure of physical healthstatus, were lower in patients with Charcot foot when compared to general population data, indicating not surprisingly poorer health status in the Charcot group. Interestingly both papers reported that the so-called ‘mental component summary scores’ (MCS) which cover aspects of mental health were not different in patients with Charcot foot compared to the general population [33,34]. In addition the mean mental and physical component summaries were not affected by gender, ethnicity and Charcot stage [33]. However, neither of the studies assessed anxiety and depression levels; we interpret these negative findings as reflecting the lack of suitability of the SF-36 as an instrument for adequately assessing mental health in this group of individuals.

## Conclusion

Anxiety and depression problems are more severe and more common in diabetes patients with Charcot foot. Female gender, being a member of a BME group and out of work carry additional risks. While problems around depressive symptoms are widely discussed in the general diabetes literature, anxiety is possibly insufficiently recognised. In our sample anxiety scores were particularly high for females. Given the poor outcomes in patients with diabetes and co-morbid depression [14] and the availability of effective psychological interventions for patients with diabetic foot complications [35], it is important to prevent and treat depression, and to review guidelines accordingly. Whether the same applies to anxiety, cannot be asserted without further studies, but similarly to depression, effective treatments exist for anxiety problems and are provided in the UK through the Improving Access to Psychological Therapies services [36]. Clinician awareness of the high risk of anxiety and depression in these mostly older and vulnerable adults [37,38] should guide appropriate screening and care planning including access to psychological support, hopefully resulting in better outcomes.

## Competing interests

The authors declare that they have no competing interests.

## Authors’ contribution

ZC developed the idea for the study, collected all the data and road an initial report as part of her MSc dissertation which was supervised by JH. CH and ZC jointly produced the initial manuscript. CH updated literature searches as part of producing the manuscript. JH produced the figures, carried out comparison analysis with other HADS data and revised the manuscript. All authors read and approved the final manuscript.
